# Multidrug resistant tuberculosis (MDR-TB) in Eritrea: a retrospective cohort analysis of mortality and associated factors (2013 – 2023)

**DOI:** 10.3389/fcimb.2026.1793864

**Published:** 2026-07-20

**Authors:** Hagos Andom Teumezgi, Nesredin Nuru Suud, Yohannes Mehari Berhane, Saleh Mohammed Said, Daniel Tesfagherghish Hailu, Kidane Kifle Habtetsion, Tekie Merawi Weldetnsae, Oliver Okoth Achila

**Affiliations:** 1 Research Unit, National Health Laboratory, Ministry of Health (MOH), Asmara, Eritrea; 2 National Tuberculosis (TB) reference Laboratory, National Health Laboratory, Ministry of Health (MOH), Asmara, Eritrea; 3 National Health Laboratory, Ministry of Health (MOH), Asmara, Eritrea; 4 Unit of Clinical Laboratory Sciences, Orotta College of Medicine and Health Sciences, Asmara, Eritrea

**Keywords:** anemia, Eritrea, MDR-RR/TB, multidrug-resistant tuberculosis, mycobacterium tuberculosis, thrombocytopenia

## Abstract

**Background:**

Multi-drug resistant tuberculosis (MDR-TB) continues to be a pressing global health concern, indiscriminate of country border and defined by higher mortality and higher treatment costs. Here, we sought to describe the MDR/RR-TB patients’ clinical profile and outcomes in Eritrea. In particular, focus was directed at hemato-biochemical predictors of mortality.

**Methods:**

This study was a retrospective (2013–2023) analysis of patients records at the Merhano MDR/RR-TB hospital in Asmara, Eritrea. In total, records from 257 patients were reviewed. Data on treatment outcomes, hematological, biochemical and demographic characteristics was subsequently abstracted using a structured check-list. We incorporated Kaplan-Meier curve and multivariate Cox regression model to evaluate the relationship between covariates and mortality.

**Results:**

The mean age (± Standard deviation (SD)) at enrolment was: 41.7 years (± 16.5), and the number of males in the cohort was 157(61.1%). Of all patients, 91(37.80%) had anemia (severe anemia, 18(7.5%); 205(79.8%) experienced adverse drug reaction (ADR); 30 (12.1%) had HIV; 45 (20.4%) had eGFR _Creat_ < 60 mL/min; 27(11.6%) had elevated BUN; 33(14.5%) had APRI score > 0.5; 45(21.6%) had hypothyroidism; 14 (5.9%) had thrombocytopenia; and 126(53.4%) had thrombocytosis. During treatment, 162 (63.0%, 95% CI: 57 – 69.6%) patients were cured, 45(17.5%, 95% CI: 13.5 – 22.0%) completed treatment, 40 (15.6%, 95% CI: 10.5-19.4%) died, and 8(3.1%, 95% CI: 1.2 – 5.1%) were Lost to follow up. Median (IQR) time to death was 33 days (10–130 days). After 137–321 person days follow up (PDFU), the incidence of death (95% CI) was 2.91(2.11 – 4.11) per 10–000 PDFU. Predictors of mortality included age >60 years; Weight <30 Kg at baseline; Thrombocytopenia; Severe anemia; APRI score > 0.5; Hypothyroidism; elevated BUN, high serum creatinine (SCr) levels; and eGFR _Creat_ < 60 mL/min. In the multivariate Cox regression model, a unit increase in hemoglobin concentration and weight reduced the risk of death by 0.79(95% CI: 0.67 – 0.93), P = 0.005 and 0.96(95% CI: 0.91-1.01), respectively. Further, higher likelihood of death was associated with elevated SCr (>12 mg/dL) levels (aHR = 6.33 (95% CI: 2.33 – 17.19), P<0.001, and APRI Score >0.5 (aHR = 2.78(95% CI: 1.65 – 4.70), P<0.001. In contrast, a unit increase in age increased the risk of death (aHR = 1.045(95 CI%: 1.02 – 1.07), P<0.001.

**Conclusion:**

The magnitude of death and other unfavorable treatment outcome for MDR/RR-TB was low. Of greater concern, however, was the critical condition of patients at presentation. Proactive strategies are needed to improve early detection of MDR/RR-TB and supportive care in patients with severe complications. In addition, additional research on MDR/TB-mortality is needed, especially among vulnerable subpopulations.

## Introduction

Tuberculosis (TB), an infection associated with the Mycobacterium tuberculosis complex (MTBC), remains a major cause of morbidity and mortality globally (Dean et al., 2022). In 2022, for instance, the World Health Organization (WHO) Global Tuberculosis Report estimated that 10.6 million people [95% uncertainty intervals (UIs): 9.9–11.4 million] developed TB and that 1.30 million people died of the disease (World Health Organisation, 2024). The reported deaths were partly attributed to multidrug-resistant (MDR) strains of TB—specifically rifampicin-resistant TB (RR-TB) and those resistant to both rifampicin and isoniazid (INH) (MDR-TB)—collectively designated as MDR/RR-TB (WHO, 2021). Other forms of TB implicated included extensively drug-resistant TB (XDR-TB) or pre-extensively drug-resistant TB (pre-XDR-TB) (WHO, 2021). Highlighting the threat posed by these organisms, the 2022 Global TB report estimated that there were 410, 000 (95% UI: 370, 000–450, 000) new cases of MDR/RR-TB globally (World Health Organisation, 2024).

In terms of deaths, MDR/RR-TB was associated with 160, 000 (95% UI: 98, 000–220, 000) deaths in 2022 (World Health Organisation, 2024). More granular data—particularly robust simulations leveraging information in the Global Burden of Disease (GBD) database—have projected that age-standardized incidence rates (ASIRs) and age-standardized mortality rates (ASMRs) for MDR-TB will marginally increase from 2022 to 2035 (Zhang et al., 2024). Most importantly, some authors have reported that MDR/RR-TB has substantial long-term health impacts and is an important contributor to Post-TB Lung Disease (PTLD), a post-TB sequela responsible for disability and suffering that often requires rehabilitation (Tiberi et al., 2019; Visca et al., 2019; Akkerman et al., 2020; Mpagama et al., 2021). More recently, some mathematical models have estimated that MDR/RR-TB was responsible for 6.9 million (95% UI: 5.5–8.5 million) disability-adjusted life-years (DALYs) (Menzies et al., 2023).

More concerning still, experts warn that if current trends persist, achieving the WHO End TB Strategy—a 90% reduction in TB mortality, an 80% reduction in incidence from 2015 levels, and the elimination of catastrophic costs for TB-affected households by 2035—will be difficult (Kendall et al., 2017; GBD 2019 Tuberculosis Collaborators, 2022). This challenge is further undermined by a multiplicity of highly interconnected factors, including the complexity of MDR/RR-TB treatment, health system capacity in low- and middle-income countries (LMICs), deficiencies in laboratory infrastructure, poor infection control, improper treatment, and cutbacks in donor support for TB programs, among others (Biagdglegne et al., 2014; Perumal et al., 2018).

Unlike drug-susceptible TB (DST), MDR/RR-TB has a longer treatment duration, higher costs (US$400–600 per person vs. US$50 per person for DST), a large pill burden, and a high likelihood of adverse drug reactions (ADRs) (World Health Organisation, 2024). Another well-documented issue is the problem of underdiagnosis and the attendant lack of data (WHO, 2021; World Health Organisation, 2024). In its latest report, the WHO noted that only a fraction of persons with MDR/RR-TB are diagnosed and that only about 2 in 5 persons accessed treatment in 2023 (World Health Organisation, 2024). The large pool of untreated patients undermines the current “treatment as prevention” strategy and adds to overall treatment costs. Further, global shocks, including the COVID-19 pandemic, resulted in a significant drop in TB case notifications (7.1 million in 2019 to 5.8 million in 2020) (Dean et al., 2022) and increased TB-related mortality (Motta et al., 2020).

In Eritrea, a country with declining TB notification rates (~65/100, 000 population vs. 300/100, 000 population for Africa), MDR/RR-TB remains a public health threat (WHO/AFRO, [[NoYear]]). Similar to other countries in the region, data on MDR/RR-TB can be described as unreliable, with estimates relying on modeling methods (WHO/AFRO, [[NoYear]]). Despite these weaknesses in data quality, the best available information points to a slightly lower disease burden. For example, a national survey conducted in May 2018 using a 100% sampling approach involving 77 microscopy centers in the country indicated that the prevalence of MDR/RR-TB among new and re-treated cases was 2.44% (95% CI: 1.22–4.32%) and 8% (95% CI: 2.22–19.23%), respectively (Mesfin et al., 2021), translating into an average of 72 cases annually (WHO/AFRO, [[NoYear]]). Beyond the small number of studies or program reports on MDR/RR-TB, little is known about this form of TB in Eritrea. In this study, we aimed to assess multiple aspects of MDR/RR-TB in the country. In particular, focus was directed at hemato-biochemical predictors of mortality.

## Methods

### Study design

We conducted a retrospective analysis utilizing data on successive cohorts of patients treated for MDR/RR-TB at the Merhano MDR-TB Hospital (period 2013 – 2023) Asmara, Eritrea. In reporting this study, we employed the Strengthening the Reporting of Observational studies in Epidemiology (STROBE) guidelines (Vandenbrouke et al., [[NoYear]]).

### Study settings

**Figure 1:**
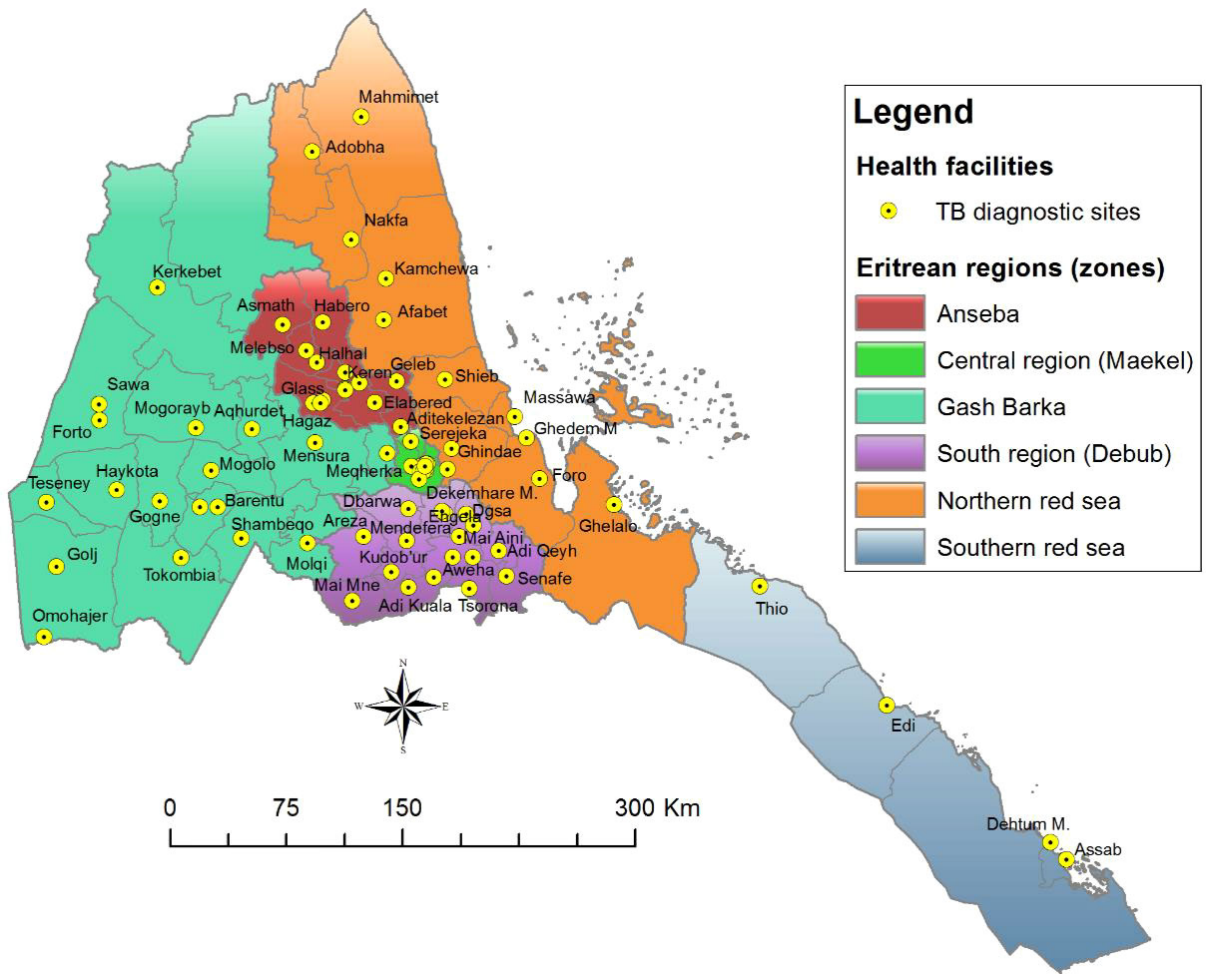
Map indicating tuberculosis laboratories in Eritrea (Adopted from NTLCP-Guideline 2020).

Eritrea, a country in the Horn of Africa, covers an area of 122, 000 Km^2^. The country is divided into six administrative regions (Zobas): Anseba, Debub, Gash-Barka, Maekel, and South Red Sea (SRS), North Red Sea (NRS). The regions are in turn subdivided into 58 sub-zobas and 699 administrative areas. Unlike other countries in the region, health care services are provided *gratis* or at a highly subsidized cost through 340 health facilities (mostly public). The facilities are ranked using a three-tier system namely, primary, secondary and tertiary. Although there is a national secretariat which manages the entire health system, the country has a number of vertical programs. For example, oversight of TB control program is implemented by National Tuberculosis and Leprosy Control Program (NTLCP) – a unit of Communicable Disease Control Division (CDC). The NTLCP has facilitators at disparate tiers of government: TB coordinators - at the zoba level; TB focal persons – in the 58 sub zobas; TB focal person – at the facility level. The programme activities at the community level is facilitated by Directly Observed Treatment of Short courses (DOTS) promoters who assist in identification and referral of suspected TB cases. Additional roles include the provision of support in treatment of diagnosed TB cases [Tuberculosis Epidemiological Review, 2019]. To facilitate prompt diagnosis of TB, NTLCP runs 76 sputum smear microscopy laboratories (**Figure 1**) - 28 facilities are equipped with GeneXpert System (Xpert MTB/RIF, Cepheid, USA) machines and the ministry maintains a mobile TB laboratory for outreach programs. In addition, sputum is routinely collected from facilities without GeneXpert expert or on-site TB microscopy laboratories to facilities with better resources.

### MDR/RR-TB treatment in Eritrea

MDR/RR-TB treatment in Eritrea is highly centralized. Existing NTLCP policy advocates for the mandatory hospitalization of confirmed cases (in the intensive phase) at the Merhano MDR/RR-TB hospital in Asmara. Established in June 23 2011, Merhano MDR/RR-TB hospital has a 50-bed capacity, an in-house laboratory equipped to perform sputum smear microscopy and routine laboratory services. However, culture (Growth on solid media (Löwenstein Jensen; Middlebrook 7H09); drug sensitivity testing (DST) – Gene X-pert or Line Probe Assay (LPA) (MTBDR *plus*, Hain Life science, Nehren, Germany); clinical chemistry (Roche-411); and hematological analysis (Bechman, DXH500) are conducted at the National Health Laboratory (NHL) where a TB reference laboratory is domiciled.

Although the country has recently adopted the 9-months short all oral treatment regime (The 6-months bedaqualine (Bdq), protamanid (Pa), linezolid (Lzd) and moxifloxacin (Mfx) (BPaLM/BPaL) MDR/RR-TB regimen has not been adopted) (World Health Organization, 2024); data for this study was collected when the standard 18–24-month regimen guideline which uses a mixed model of care comprising of an intensive phase and a continuation phase was in place. In the intensive phase (mandatory 6–8 months hospitalization phase), patients were placed on regimens of p-aminosalicylic acid (PAS), Kanamycin (Km), Levofloxacin (Lfx), Ethionamide (Eto), Cycloserine (Cs). In the continuation phase, patients are referred to the nearest health facility for treatment administration or to community-DOTs providers (Araia et al., 2021). The regimen in this period comprises largely of 12 months of PAS, Lfx, Eto, and Cs. During the continuation phase, patients are required to attend the National RR/MDR-TB hospital at Merhano or respective zonal referral hospital for their monthly follow-up. A small emolument (~100 USD per month) is usually given to patients during visits. Per protocol weight, monthly sputum samples, clinical chemistry, and hematological measurements are routinely measured. In addition, patients are required to visit the national RR/MDR-TB at the end of the continuation phase, in case of treatment failure, or presence of significant complications or side effects (Mesfin et al., 2021).

### Study Participants

Patient with MDR/RR-TB, confirmed by either GeneX-pert/LPA or culture, who received treatment at the Merhano MDR-TB hospital in the period spanning 2013–2023 were included in the study. Additional inclusion criteria included patients with data on treatment outcome. Exclusion criteria included absence of Gene X-pert/LPA or culture data and absence of sputum culture data in the first two- three months of treatment.

### Data source and data collection and reporting system for MDR-TB patients

**Figure 2:**
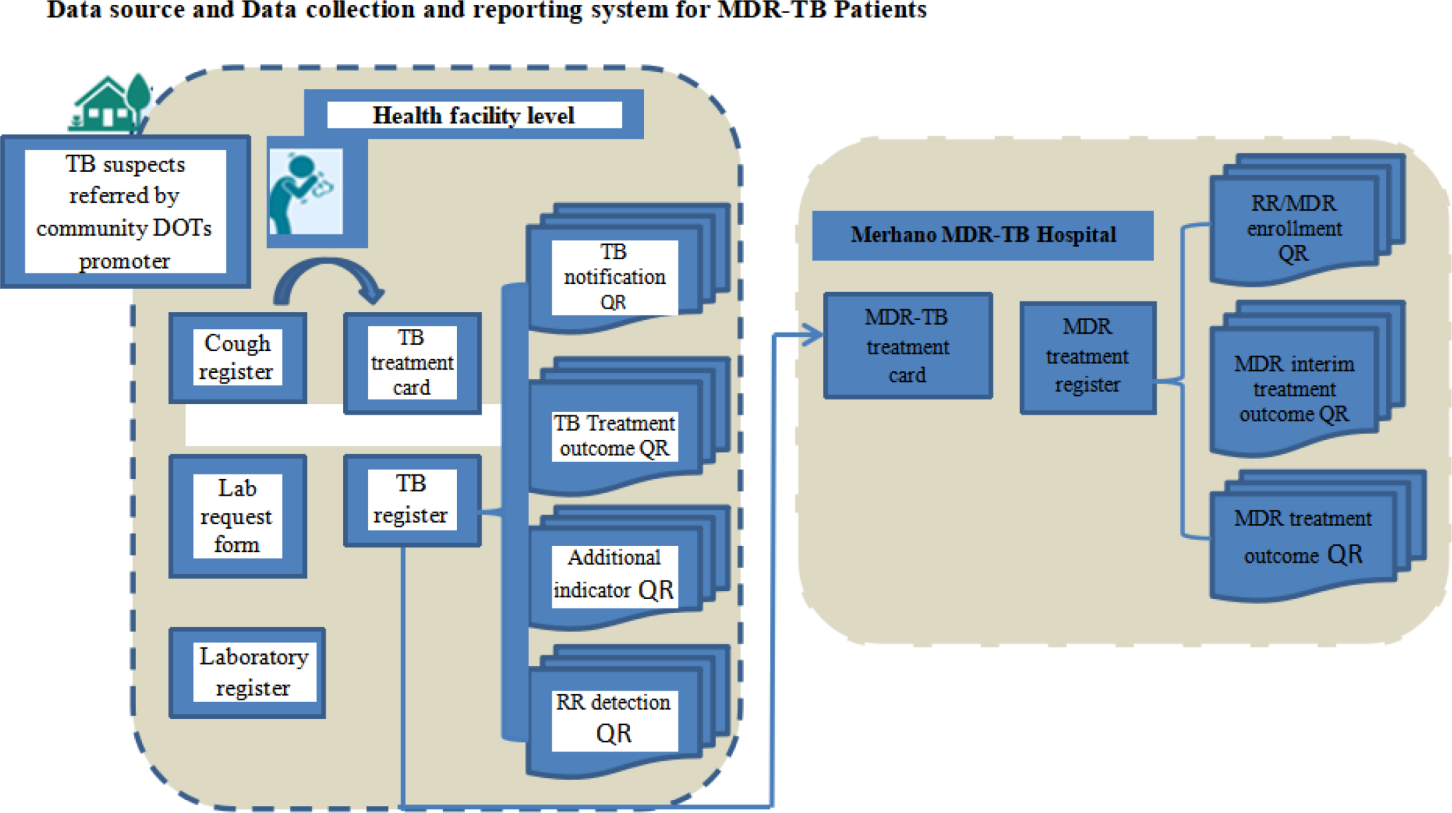
Data source and data collection and reporting system for MDR-TB patients.

In Eritrea, TB patients come into contact with formal care in multiple ways. First and the most direct pathway is where patients make direct contact with care centers. An alternative pathway is where suspected TB patients are referred to health facilities by community DOTS promoters. Regardless of the manner of contact, presumptive TB cases are all registered in the Cough Register. After sputum sample collection, Sputum Request Form is completed, this document accompanies the sample to the laboratory. The sputum request form has two sections; an upper portion which includes basic demographic and contact details of the patient being tested and a lower section which is used to communicate results back to the facility that requested the tests. All tests performed in the laboratory are recorded in TB Laboratory Register. Once a patient diagnosed with TB, their details are recorded in the TB Register – The register is used to monitor program performance, summarize testing results, treatment decisions, and develop several quarterly reports (**Figure 2**). Patients with MDR/RR-TB are subsequently referred for treatment at the Merhano MDR-TB hospital. At the hospital, patient’s details are registered in the MDR-TB treatment card and the MDR-treatment register. The latter document is subsequently used to develop several reports - MDR/RR-TB treatment outcome quarterly reports (QR), MDR interim outcome QR, and MDR-enrolment QR) (WHO/AFRO, [[NoYear]]).

In this study, information on MDR/RR-TB patients was collected from the MDR-TB register. Using specifically designed data collection forms, information on demographic factors, clinical information and patient’s current status (dead, LTFU, transferred out (TOs), treatment completed, and treatment failure) were systematically collected by members of the research group. Team members responsible for collecting and entering study data into appropriate digital formats (Excel) received appropriate training and supervision. More importantly, the data was routinely assessed for completeness and consistency by principal investigators and co-investigators.

### Treatment and outcome measures

Three outcomes measures (time-to-death, cure or treatment completion) were evaluated. However, mortality was the principal outcome measure. Additional patient level covariates that may also affect survival or mortality were examined, including gender (male and female); age at enrollment; region (Zoba); address (urban and rural); weight, HIV status, TB resistotypes (RR only of RI(MDR)); adverse drug reaction (ADR); type of patients (New, Relapse, and retreatment after failure); Liver function tests (LFTs) (ALT and AST); kidney panel (Serum creatinine (SCr), Blood Urea Nitrogen (BUN)); Thyroid function tests (TSH, T_3_ uptake, T_4_); and hematological indexes (hemoglobin, hematocrit, Red Blood Cells (RBCs), platelets (PLT), White Blood Cells (WBC). Composite measurements such as AST/PLT ratio (APRI) score and Creatinine-based estimated glomerular filtration rates (eGFR _Creat_) were also evaluated. APRI score was evaluated using the following formula: APRI = [(AST/upper limit of the normal AST level) × 100]/PLT (× 10^9^/L) (Sarin et al., 2016). eGFR _Creat_ was estimated using Cockcroft-Gault (CG) equation (mL/min) = (140 – Age) × Weight (kg)/72 x S_cr_ (mg/dL) x (0.85 if female) (Levey et al., 2006). The use of CG for eGFR _Creat_ estimation, as opposed to options such as the Modification of Diet in Renal Disease (MDRD) or Chronic Kidney Disease Epidemiology Collaboration (CKD-EPI); was informed by several considerations. Foremost was the fact that CG equation is preferred by regulatory urgency agencies such as Federal Drug Agency (FDA) (U.S. Food and Drug Administration (FDA), 2010). This preference is based on the fact that options like MDRD formula or CKD-EPI are normalized for body surface (BSA) making them less reliable for drug dosing in underweight and overweight patients (U.S. Food and Drug Administration (FDA), 2010). The degrees of anemia were classified according to the WHO criteria: mild anemia: 11.0 - 12.9 g/dL (men) and 11.0 - 11.9 g/dL (women), moderate anemia: 8.0 - 10.9 g/dL (both sexes), and severe anemia: < 8.0 g/dL (both sexes) (World Health Organisation, 2011). Thrombocytosis and thrombocytopenia were defined as PLT count > 450 X 10^9^/L and PLT < 150 x 10^9^/L respectively. Hypothyroidism was defined as TSH > 4.5 (μU/L) (subclinical hypothyroidism > 4.5 – 10 (μU/L); Overt hypothyroidism > 10 (μU/L)) (Garber et al., 2012).

### Operational definitions for study outcomes

For these analyses, respective outcomes were defined as follows:

1. Death: defined as all-cause mortality occurring during the patient’s follow-up (Umanah et al., 2015).
2. Cured: TB patient whose sputum is bacteriologically-confirmed at the beginning of treatment and who was smear- or culture-negative in the last month of treatment and on at least one previous occasion (Demile et al., 2018).
3. Loss-to-follow-up (LTFU) was defined as non-attendance for 2 consecutive months of scheduled clinic appointments after enrollment into care (Woldeyohannes et al., 2019). For institutionalized in-patients in the intensive phase of MDR-TB treatment, it was defined as those who escaped or left care without due authorization before completing treatment.
4. Treatment completed (TCs): A TB patient who completed treatment without evidence of failure but there is no record to show that sputum smear grade or culture results in the last month of treatment and on at least one previous occasion are negative, either because they were not undertaken or because results were not documented (Demile et al., 2018).

### Data processing and analysis

Analysis was conducted using IBM SPSS (version 26) and STATA version 12.0 (STATA Corporation, College Station, TX). Depending on data type, summaries were undertaken using counts (frequency), proportions (percentages), means (± SD) and medians (interquartile range (IQR). Where appropriate, Chi-Squire (χ^2^) and Fishers exact test; parametric (ANOVA and t-test); and nonparametric statistics (Mann-Whitney U and Kruskal Wallis) were used to evaluate differences. Incidence of mortality was calculated per 10–000 person days (PD) (95% CI). Kaplan-Meier (KM) curves and associated Log- rank (Mantel-Cox) tests were conducted to assess time variance in mortality by specific clinical and demographic variables. The Multivariate Cox regression model was implemented to assess the variables that predict mortality. Variables with a P<0.25 in the bivariate analysis were incorporated in the model. Log - log plots and plots of Schoenfeld residuals were used to evaluate the proportional hazard assumptions. The final results are presented as an adjusted or unadjusted hazard ratio with a 95% confidence interval (CI). A two-sided p-value <0.05 was considered significant.

## Results

From 2013-2023, information on 257 patients with MDR/RR-TB was available in records obtained from Merhano MDR-TB hospital. In terms of regional distribution, 65(25.3%) were from Maekel, 65(25.3%) from Debub, 51(19.8%) from Gash-Barka, 35(13.6%) from Anseba, 35(13.6%) from Northern Red Sea (NRS) and 6(2.3%) from the Southern Red Sea (SRS). Among cases registered during the study period, median (IQR) delay between the laboratory diagnosis and treatment initiation was 3 days (IQR: 1–6 days).

### Baseline sociodemographic and clinical characteristics of patients with MDR-TB in Eritrea

The mean age (± SD) at enrolment was 41.7 years (± 16.5) with a higher number of males (males = 157(61.1%) vs Females = 100(38.9%)). No change in the mean (± SD) ages was observed overtime (e.g. mean age from <2015, 2016 – 2018, 2019 – 2021, and > 2021 were 40.9 years (± 15); 40 years (± 15.9); 44(± 18.9) years; and 42.4(± 16) years, respectively, P = 0.54. In a separate RR vs RI MDR-TB analysis, the respective proportions were 223(87.1%) and 33(12.9%) with New, Relapse, and Treatment after failure constituting 82(32.4%), 12(4.7%) and 159(62.8%) of the cases, respectively. Mean weight at diagnosis was 43.81 (± 10.22) Kg, and proportions of patients weighing < 30 Kg; 30–40 Kg; 41–50 Kg; and >51 kg were 17(6.6%), 95 (33.1%), 97(37.7%) and 58 (22.6%), respectively. Further, overall prevalence of anemia was 91(37.80). In terms of anemia severity, the respective proportions were severe anemia, 18(7.5%); moderate anemia, 27 (10%), mild anemia, 49(20.3%). Of all patients, 30 (12.1%) were found to HIV Positive and 205(79.8%), experienced adverse drug reaction (ADR). In addition45 (20.4%) had eGFR _Creat_ < 60 mL/min, 45(21.6%) had hypothyroidism (30(14.4%) had subclinical hypothyroidism and 15(7.2%) had overt hypothyroidism), 14 (5.9%) had low PLT count, 126(53.4%) had high PLT count and 33(14.5%) had APRI score > 0.5. Disaggregation of APRI score with respect to fibrosis/cirrhosis strata indicated that 190(85.2%) had APRI score <0.5 indicating little or no fibrosis category (F0-F1 on METAVIR); 26 (11.7) had APRI score < 0.5 – 1.5) corresponding to mild to moderate fibrosis (F1-F2 on METAVIR); 3(1.3%) had APRI score of >1.5–2 which is associated with advanced fibrosis (F3-F4 on METAVIR) and 4(1.8%) had APRI scores > 2 which is strongly associated with cirrhosis (F4 on METAVIR). See **Table 1** for more information.

**Table 1:** Distribution of specific demographic and clinical variables across year bands.

Cohort characteristics	<2015		2016 - 2018	2019 - 2021	>2022	Statistical test	Population
Total	91(35.4)		62(24.1)	69(26.8)	35(13.6)		257
Gender							
Male	48(52.7)		42(67.7)	44(63.8)	23(65.7)	0.227 (4.34)	157(61.1)
Female	43(47.3)		20(32.3)	25(36.2)	12(34.3)		100(38.9)
Age at enrolment in years	40.9 (± 15)		40 (± 15.9)	44(± 18.9)	42.4(± 16)	0.54(0.72)^a^	41.7(± 16.5)
<40	48(52.7)		34(54.8)	38(55.1)	17(48.6)	0.487 (5.45)	137(53.3)
40 - 60	31(34.1)		18(29.0)	15(21.7)	13(37.1)		77(30.0)
>60	12(13.2)		10(16.1)	5(23.2)	5(14.3)		43(16.7)
Region (Zoba)							
Maekel	24(26.4)		11(17.7)	18(26.1)	12(34.3)	0.088 (22.81)	65(25.3)
Debub	28(30.8)		17(27.4)	15(21.7)	5(14.3)		65(25.3)
Gash-Barka	17(18.7)		17(27.4)	12(17.4)	5(14.3)		51(19.8)
Anseba	13(14.3)		10(16.1)	8(11.6)	4(11.4)		35(13.6)
Northern Red Sea	7(7.7)		7(11.3)	15(21.7)	6(17.1)		35(13.6)
Southern Red Sea	2(33.3)		0(0.0)	1(1.4)	3(8.6)		6(2.3)
Address							
Urban	54(59.3)		30(48.4)	38(55.1)	20(57.1)	0.604 (1.85)	142(55.3)
Rural	37(40.7)		32(51.6)	31(44.9)	15(42.9)	115(44.7)
Wt at diagnosis (Kg)	43(± 10.5)		44.3(± 10.1)	44(± 10.3)	45(± 9.6)		44(± 10.2)
<30	7(7.7)		4(6.5)	4(5.8)	2(5.7)	0.933 (3.65)	17(6.6)
30- 40	32(35.2)		19(30.6)	26(37.7)	8(22.9)		85(33.1)
41 - 50	34(37.4)		25(40.3)	23(33.3)	15(42.9)		97(37.7)
>51	18(19.8)		14(22.6)	16(23.2)	10(28.6)		58(22.6)
HIV status							
Negative	76(84.4)		53(91.4)	57(89.1)	31(88.6)	0.624 (1.76)	217(87.9)
Positive	14(15.6)		5(8.6)	7(10.9)	4(11.4)		30(12.1)
Type of MDR/RR patient							
RR only	62(68.1)		58(95.1)	69(100)	34(97.1)	<0.001 (45.99)	223(87.1)
RI(MDR)	29(31.9)		3(4.9)	0(0.0)	1(2.9)		33(12.9)
Patience experience any ADR							
Yes	64(70.3)		62(100)	56(81.2)	23(65.7)	<0.001 (25.11)	205(79.8)
No	27 (29.7)		0(0.0)	13(18.8)	12(34.3)		52(20.2)
Type of patient							
New	4(4.5)		16(26.2)	43(63.2)	19(54.3)	<0.001 (74.9)	82(32.4)
Relapse	3(3.4)		3(4.9)	3(4.4)	3(8.6)		12(4.7)
Retreatment after failure	82(92.1)		42(68.9)	22(32.4)	13(37.1)	159(62.8)
ALT (U/L)	10(7 – 15)		11(7-20)	17(10 – 22)	19(13-29)	<0.001^k^	12(8-20)
< 40	74(98.7)		52(88.1)	57(91.9)	31(88.6)	0.084 (6.646)	194(89.8)
> 40	1(1.3)		7(11.9)	5(8.1)	4(11.4)	22(10.2)
AST	31(24 – 42)		28(22 – 34)	31(25-38)	26(21-36)	0.151^k^	29(23-38)
< 35	45(60.0)		47(79.9)	41(66.1)	25(71.4)	0.102 (6.206)	158 (68.4)
>35	30(40.0)		12(20.3)	21(33.9)	10(28.6)		73(31.6)
APRI Score							
<0.5	64(33.0)		47(24.2)	53(27.3)	30(15.5)	0.415 (2.851)	194(85.5)
>0.5	7(21.2)		12(36.4)	9(27.3)	5(15.2)		33(14.5)
Hemoglobin (g/dL)	13.1 (± 2.6)		13.3(± 2.5)	12.9(± 2.46)	13.4(± 2.56)	0.663^a^	13.12(± 2.53)
Anemia							
Severe anemia		11(3.9)	2(3.3)	3(4.5)	2(5.7)	0.386 (9.577)	18(7.5)
Moderate anemia		6(7.6)	8(13.1)	7(10.6)	3(8.6)		24(10.0)
Mild anemia		15(19.0)	14(23.0)	15(22.7)	5(14.3)		49(20.3)
Normal levels		47(59.5)	37(60.7)	41(62.1)	25(71.4)		150(62.2)
Hematocrit (%)		39.5(± 8.0)	41.4(± 6.6)	38.0 (± 7.1)	40(± 7.1)	0.072^a^	39.65(± 7.4)
RBC (10^6^/µL)		4.6(± 0.9)	4.7(± 0.7)	4.5(0.9)	4.62(0.9)	0.292^k^	4.6(± 0.9)
Platelets (×10^9^/L)		372(308-537)	348(234 – 441)	347(247-471)	387(336-500)	0.105^k^	360(279-479)
Platelets categories							
Thrombocytopenia		2(2.7)	6(10.0)	4(6.1)	2(5.7)	0.476 (5.546)	14(5.9)
Normal		30(40.0)	25(41.7)	30(45.5)	11(31.4)		96(40.7)
Thrombocytosis		43(57.3)	29(48.3)	32(48.5)	22(62.9)		126(53.4)
WBC x 10^9^/L		7(	7	6.5	6	0.104^k^	7(5-9)
TSH ((μU/L)		4.0(2-6)	4(2.2-6.3)	2.6(1.8-3.6)	1.6(1.2-2.5)	<0.001^k^	2.9(1.6-5.0)
Thyroid disorders							
Hyperthyroidism		1(1.7)	2(3.4)	0(0.0)	0(0.0)	0.010 (16.86)	3(1.4)
Normal		41(69.5)	38(65.5)	47(83.9)	34(97.1)		160(76.9)
Hypothyroidism		17(28.8)	18(31.0)	9(16.1)	1(2.2)		45(21.6)
Total Thyroxine (T4) ng/dL							
T_3_ nmol/L		7.2(6-9)	7(5 – 9)	8(6.3-10)	7.9(7.4-9.2)	0.087^k^	7.8(6-9)
BUN (mg/dL)		12(9-13.7)	12(9.8-15.2)	12(9.2-15)	9(8-14)	0.163^k^	11(9-14)
Normal <20 mg/dL		71(94.7)	50(87.7)	56(84.8)	29(82.9)	0.190 (4.76)	206(88.4)
High >20 mg/dL		4(5.3)	7(12.3)	10(15.2)	6(17.1)		27(11.6)
SCr (mg/dL)		0.7(0.5-0.8)	0.8(0.6-0.9)	0.7(0.6-0.9)	0.6(0.5-0.7)	0.001^k^	0.7(0.5-0.8)
SCr categories							
Normal (<1.3 mg/dL)		71(94.7)	55(91.7)	62(93.9)	33(94.3)	0.906 (0.560)	221(93.6)
High (>1.3 mg/dL)		4(5.3)	5(8.3)	4(6.1)	2(5.7)		15(6.4)
Cockcroft-Gault equation eGFR							
<60 (mL/min)		11(16.2)	13(23.2)	17(26.6)	4(12.1)	0.271 (3.92)	45(20.4)
>60 (mL/min)		57(83.8)	43(76.8)	47(73.4)	29(87.9)		176(79.6)

Comparisons of proportions were performed by using the chi-squire (χ2) or Fishers exact test, Mean (± SD) by ANOVA, and median (IQR) distribution by Kruskal Wallis test.

^k^ Kruskal Wallis test.

^a^ ANOVA.

ALT, alanine aminotransferase; AST, aspartate aminotransferase; HIV, human immunodeficiency virus; RBC, Red Blood Cells; BUN, Blood Urea Nitrogen; APRI, TSH, Thyroid stimulating hormone; T3, triiodothyronine; T4, Thyroxine; Serum Creatinine (SCr); MDR, Multidrug resistance; WBC, White blood cells.

Hypothyroidism: TSH > 5.0 mU/L.

Hyperthyroidism: TSH < 0.5 mU/L.

### Prevalence and trends of mortality and other outcomes in patients with MDR-TB in Eritrea

**Figure 3:**
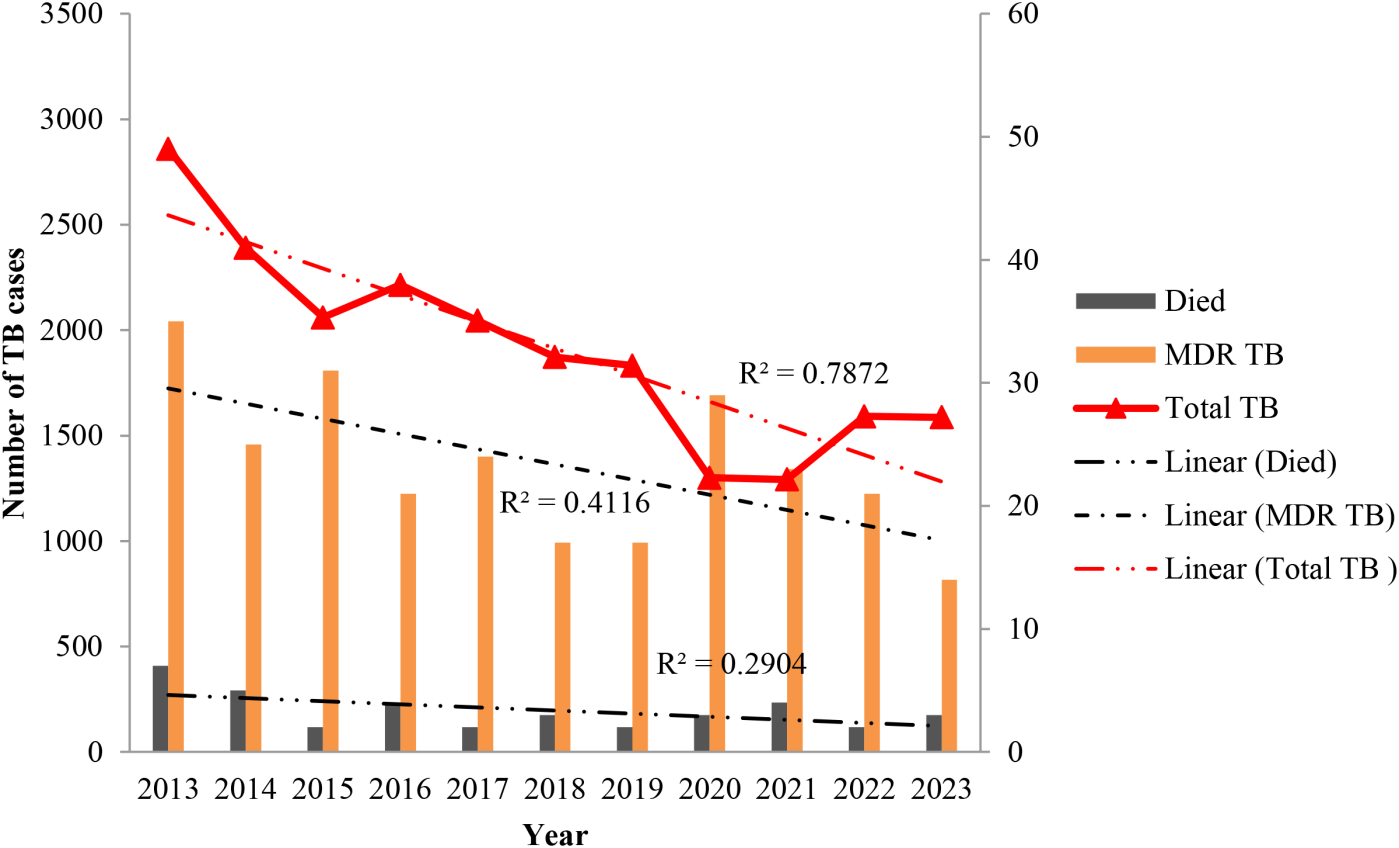
Magnitude and trends of overall TB, MDR-TB and MDR-TB associated deaths in Eritrea, 2013 – 2023.

Of the 257 patients treated at the Merhano MDR-TB hospital in the period spanning 2013 – 2023, 162 (63.0%, 95% CI: 57 – 69.6%) patients were cured, 45(17.5%, 95% CI: 13.5 – 22.0) completed treatment, 40 (15.6%, 95% CI: 10.5-19.4%) died, and 8(3.1%, 95% CI: 1.2 – 5.1%) were LTFU. Further, the treatment outcome of 2(0.8%, 95% CI: 0.0 – 2.3%) patients was designated as other (Discontinued). **Figure 3** shows overall number of TB cases in Eritrea, number of MDR-TB cases and number of deaths between 2013 – 2023. Trends and associated R^2^ are also shown. In a separate Pearson Correlation analysis, we demonstrated that the relationship between year and overall deaths, MDR-TB and overall TB cases were - 0.539 (P = 0.044), - 0.642 (P = 0.017), - 0.780 (P = 0.002), respectively, see **Figure 3**.

### Factors associated with MDR TB in Eritrea

Bivariate analysis of differences between patients with disparate treatment outcomes (cured, died, completed treatment) uncovered several significant associations including significant differences in mean age (± SD) (51.7 (± 18.3) vs 40.0 (± 15.5) years and 40(± 14.2) years in those who were cured or completed treatment, P<0.001; Weight at time of diagnosis (a lower median (IQR) in those who died: 39.0 Kg (IQR: 32-47) Kg vs 45(IQR: 38-50) Kg and 42 Kg (IQR: 37-47) Kg in those who were cured or completed treatment, P < 0.001; ALT U/L(higher in those who died =14 U/L (IQR: 8-25) U/L vs 14 U/L (IQR: 9–21 U/L) U/L and 10.5 U/L (IQR: 6-16.5(U/L) in others, P = 0.017; Hemoglobin levels (lower in those who died: 11.4 (IQR: 8.5-15) g/dL vs 13 (IQR: 12-15) g/dL in those who were cured and 13.5 g/dL (IQR: 12-16) g/dL, P = 0.004; SCr (higher in patients who died: 0.9 (IQR: 0.45-1.8) mg/dL vs 0.7 (IQR: 0.5-0.8) mg/dL in those who were cured and 0.6 mg/dL (IQR: 0.45-0.7) mg/dL in those who completed treatment, P<0.0001. Further, our analysis demonstrated that 11(42.3%) of the patients with APRI > 0.5 died. Similarly, death rate among the 4 patients in the cirrhosis likely category (APRI score > 2 or F4 on METAVIR) was 100%. Moreover, 8(48%) and 5(62.5%) of patients with BUN >20 mg/dL and 30 mg/dL died. See **Table 2** for further details.

**Table 2:** Demographic and clinical characteristic of participants stratified by survival outcome.

Cohort characteristics	Cured	Died	Treatment completed	Statistical test
Prevalence	162 (63)	40 (15.6)	45 (17.5)	
Time-to-event (Days)	731 (556-735)	33 (10-130)	734 (732-740)	<0.001^k^
Gender				
Male	104 (68)	22 (14.4)	27 (17.6)	0.127 (4.126)
Female	58 (61.7)	23 (24.5)	13 (13.8)	
Age at enrolment in years	40.0 (± 15.5)	51.7 (± 18.3)	40 (± 14.2)	<0.001^a^
<18	6 (66.7)	0 (0.0)	3 (33.3)	<0.001 (28.83)
19 - 40	94 (72.9)	14 (10.9)	21 (16.3)	
41 – 60	43 (62.3)	9 (13.0)	17 (24.6)	
>60	19 (47.5)	17 (42.5)	4 (10.0)	
Region (Zoba)				
Maekel	39 (66.1)	9 (15.3)	11 (18.6)	0.371 (10.83)
Debub	37 (59.7)	11 (17.7)	14 (22.6)	
Gash-Barka	38 (74.5)	3 (5.9)	10 (19.6)	
Anseba	20 (57.1)	9 (25.7)	6 (17.1)	
Northern Red Sea	25 (73.5)	6 (17.6)	3 (8.8)	
Southern Red Sea	3 (50.0)	2 (33.3)	1 (16.7)	
Address				
Urban	85 (63.0)	21 (15.6)	29 (21.5)	0.345 (2.127)
Rural	77 (68.8)	19 (17.0)	16 (14.3)	
Year of enrolment				
< 2015	23 (27.1)	17 (20.0)	45 (52.9)	<0.001 (116.39)
2016 - 2018	52 (85.2)	9 (14.8)	0 (0.0)	
2019 - 2021	60 (87.0)	9 (13.0)	0 (0.0)	
>2021	27 (84.4)	5 (15.6)	0 (0.0)	
Weight at time of Diagnosis (Kg)	45 (38-50)	39 (32-47)	42 (37-47)	0.001^k^
<30	9 (52.9)	6 (35.3)	2 (11.8)	0.050 (12.614)
30- 40	45 (55.6)	18 (22.2)	18 (22.2)	
41 - 50	68 (71.6)	10 (10.5)	17 (17.9)	
>51	40 (74.1)	6 (11.1)	8 (14.8)	
HIV status				
Negative	138 (66.3)	32 (15.4)	38 (18.3)	0.497 (1.40)
Positive	16 (55.2)	6 (20.7)	7 (24.1)	
Type of MDR/RR patient				
RR only	156 (72.6)	32 (14.8)	28 (13.0)	<0.001 (42.2)
RI (MDR)	5 (17.9)	8 (26.7)	17 (56.7)	
Patience experience any ADR				
Yes	144 (71.6)	25 (12.4)	32 (15.9)	<0.001 (18.56)
No	18 (39.1)	15 (32.6)	13 (28.3)	
Type of patient				
New	68 (86.1)	10 (12.7)	1 (1.3)	<0.001 (27.528)
Relapse	7 (63.6)	2 (18.2)	2 (18.2)	
Retreatment after failure	84 (54.9)	28 (18.3)	41 (26.8)	
Alanine Aminotransferase (U/L)	14 (9-21)	14 (8 – 25)	10 (6 -16.5)	0.017^k^
Normal < 35 U/L	138 (72.3)	11 (5.8)	42 (22.0)	0.080 (5.05)
High > 35 U/L	17 (81.0)	3 (14.3)	1 (4.8)	
Aspartate Aminotransferase (U/L)	28 (23 – 36)	31.2 (23 – 67)	31 (27.5 -48)	0.420
Normal (< 35 U/L)	112 (72.3)	16 (10.3)	27 (17.4)	0.133 (4.031)
High (>35 U/L)	43 (59.7)	13 (18.1)	16 (22.2)	
APRI Score	0.24 (0.16-0.3)	0.53 (0.2-0.9)	0.2 (0.16-0.32)	0.079
APRI score category				
<0.5	136 (71.6)	15 (7.9)	39 (20.6)	<0.001 (18.35)
>0.5	19 (57.6)	11 (33.3)	3 (9.1)	
Hemoglobin (g/dL)	13.0 (12-15)	11.4 (8.5-15)	13 (12-16)	0.160 ^k^
Anemia				
Severe anemia	2 (11.8)	11 (64.7)	4 (23.5)	<0.001 (48.67)
Moderate anemia	14 (58.3)	6 (25.0)	4 (16.7)	
Mild anemia	33 (68.8)	6 (12.5)	9 (18.8)	
Normal	111 (75.5)	10 (6.8)	26 (17.7)	
Hematocrit (L/L)	40.6 (6.5)	35.2 (8.5)	38.8 (8.8)	0.001^a^
RBC (10^6^/µL)	5.0 (4.0 - 5)	4 (3.79 – 5.0)	4.3 (4.0 – 5.0)	0.016^k^
Platelets (×10^9^/L)	356 (281-457)	202 (138-342)	413 (323-543)	0.003^k^
Thrombocytopenia	6 (42.9)	6 (42.9)	2 (14.3)	0.004 (15.643)
Normal	69 (73.4)	12 (12.8)	13 (13.8)	
Thrombocytosis	85 (68.5)	11 (8.9)	28 (22.6)	
White Blood Cells (WBC) (x10^9^/L)	7 (5-9)	6.2 (4.2-9.0)	7 (6.0-9.5)	0.104
TSH (μU/L)	2.7 (1.4-4.2)	3.1 (2.2-9.1)	3.9 (1.9-6.5)	0.249
Thyroid disorders				
Hyperthyroidism	1 (33.3)	1 (33.3)	1 (33.3)	0.045 (9.735)
Normal	122 (78.2)	14 (9.0)	20 (12.8)	
Hypothyroidism	27 (60.0)	10 (22.2)	8 (17.8)	
Total T4 ng/dL	8 (6-10)	7 (5-8.6)	7.5 (5.3-9)	0.113^k^
T3 uptake (nmol/L)	1 (1.0-1.1)	0.90 (0.8-1.0)	0.94 (0.9-1.1)	<0.001^k^
BUN (mg/dL)	11 (9.0 -14.0)	18 (10.5-26)	11 (9-14)	<0.001^k^
<20 mg/dL	144 (71.3)	16 (7.9)	42 (20.8)	<0.001 (36)
>20 mg/dL	13 (48.1)	13 (48.1)	1 (3.7)	
SCr (mg/dL)	0.7 (0.5-0.8)	0.9 (0.45-1.8)	0.6 (0.45-0.7)	<0.001^k^
<1.3 mg/dL	154 (71.0)	21 (9.7)	42 (19.4)	<0.001 (24.56)
>1.3 mg/dL	6 (40.0)	8 (53.3)	1 (6.7)	
Cockcroft-Gault equation eGFR (mL/min)				
< 60 (mL/min)	33 (73.3)	8 (17.8)	4 (8.9)	0.003 (11.55)
> 60 (mL/min)	126 (73.3)	8 (4.7)	38 (22.1)	

Comparisons of proportions were performed by using the chi-squire (χ2) or Fishers exact test, Mean (± SD) by ANOVA, and median (IQR) distribution by Kruskal Wallis test.

^k^ Kruskal Wallis test.

^a^ ANOVA.

Interquartile range.

ALT, alanine aminotransferase; AST, aspartate aminotransferase; HIV, human immunodeficiency virus; RBC, Red Blood Cells; BUN, Blood Urea Nitrogen; APRI, TSH, Thyroid stimulating hormone; MDR, Multidrug resistance; SCr, Serum Creatinine; T3, triiodothyronine; T4, Thyroxine, RI, Rifampicin; INH, Isoniazid.

### Incidence of mortality stratified by selected variables in patients with MDR-TB in Eritrea

After 137–321 person days follow up (PDFU), the incidence of death (95% CI) was 2.91(2.11 – 4.11) per 10–000 PDFU. The high incidence of mortality was associated with the following: age >60 years: 9.76 (5.89-16.2) per 10–000 PDFU; Weight <30 Kg at the time of diagnosis: 8.78(3.94– 19.5) per 10–000 PDFU; Low PLT count (<150 x 10^9^/L): 5.01(1.62 – 15.5) per 10–000 PDFU; Severe anemia (Hg < 8 (g/dL): 11.4(4.77 – 27.51) per 10–000 PDFU; APRI score > 0.5: 4.23(2.02 – 8.88) per 10–000 PDFU; High TSH (>5 μU/mL): 3.0(1.57 – 5.43) per 10–000 PDFU; High SCr: 10.1(4.81 – 21.18) Per 10–000 PDFU; low eGFR _Creat_: 3.34(1.69-7.59) per 10–000 PDFU; and high BUN: 13.4(7.8-23.06) per 10–000 PDFU as it can be seen in **Table 3**.

**Table 3:** Incidence of death and mean survival duration in days for patients with MDR TB in Eritrea (2013 – 2023).

Cohort Characteristics	Incidence of death per 10–000 person days (95% CI)	Mean for survival time in days (95% CI)	P value Log rank (Mantel-Cox)
Overall	2.91 (2.11-4.11)	684 (650-717)	
Gender			
Male	3.28 (2.21 – 5.00)	676.0 (632 -719)	0.440 (0.595)
Female	2.37 (1.35 – 4.46)	669 (618 – 7215)	
Age at enrolment in years			
< 40	1.58 (0.89 – 3.03)	715 (677-753)	<0.001 (24.01)
41 – 60	2.49 (1.36 –5.00)	697 (640-754.6)	
>60	9.90 (5.7 – 17.6)	512 (406-618)	
Address			
Urban	2.65 (1.70 – 4.30)	691 (647 – 735)	0.652 (0.203)
Rural	3.27 (2.04 – 5.46)	664 (612 –716)	
Year of enrolment			
< 2015	3.39 (2.04 -5.93)	628 (566 – 690)	0.595 (1.891)
2016 - 2018	2.48 (1.26 -5.38)	693 (628 – 759)	
2019 - 2021	2.40 (1.25-5.12)	668 (618-719)	
>2021	3.69 (1.48-11.10)	493 (427 – 558.8)	
Weight at time of diagnosis (Kg)			
<30	8.77 (3.24– 25.7)	473 (301 – 646)	0.017 (10.173)
30- 40	3.93 (2.41 – 6.7)	626.7 (566 – 687)	
41 - 50	1.85 (0.98 – 3.38)	693 (647 – 739)	
>51	1.96 (0.87 – 5.16)	726 (668 – 785)	
HIV status			
Negative	2.72 (1.9-3.99)	691 (656 –727)	0.474 (0.513)
Positive	3.82 (1.6-10.49)	607 (503 – 712.2)	
Type of MDR/RR patient			
RR only	2.66 (1.86-3.90)	695 (661 – 729)	0.061 (3.50)
RI (MDR)	4.93 (2.25-11.81)	579 (462 – 696)	
Type of patient			
New	2.31 (1.24 – 4.29)	670 (647 – 751)	0.548 (1.205)
Relapse	3.16 (0.63 – 28.85)	657 (482 – 832.4)	
Retreatment after failure	3.25 (2.20 – 4.95)	668 (623 – 713)	
Alanine Aminotransferase (U/L)			
< 40 U/L	2.04 (1.37-3.15)	715 (684 – 748)	0.143 (2.148)
> 40 U/L	5.04 (1.72-18.1)	581 (439 – 723)	
Aspartate Aminotransferase (U/L)			
Normal (< 35 U/L)	1.72 (1.05 – 2.97)	731 (698 – 764)	0.076 (3.14)
High (>35 U/L)	3.38 (1.90 –6.42)	638 (572- 704)	
Platelets (x10^9^/L)			
Thrombocytopenia	9.9 (3.57 – 29.2)	474 (275 – 674.2)	<0.001 (15.63)
Normal	2.22 (1.25-4.26)	710 (662 – 760)	
Thrombocytosis	1.50 (0.8 – 2.96)	711 (676 – 745)	
Anemia			
Severe anemia	23.2 (9.31 – 55.6)	314.1 (154 – 475.4)	<0.001 (60.61)
Moderate anemia	4.65 (1.94 -12.69)	580 (462 – 697)	
Mild anemia	2.0 (0.9 –5.34)	687 (624 – 748.2)	
Normal	1.2 (0.6 – 2.37)	753 (724 – 782)	
APRI Score			
<0.5	1.31 (0.79-2.3)	747 (720 – 774)	<0.001 (18.6)
>0.5	6.91 (3.51 – 14.34)	554 (438 – 670.9)	
Thyroid stimulating hormone (TSH)			
Normal	1.58 (0.9 – 2.86)	723 (689 – 757)	0.040 (6.46)
Hypothyroidism	3.85 (2.01-7.98)	652 (567 – 736)	
Cockcroft-Gault equation eGFR			
<60 mL/min	3.34 (1.69-7.59)	664 (591-736)	0.001 (10.197)
>60 mL/min	0.74 (0.4-1.67)	768.8 (747-790.7)	
BUN			
<20 mg/dL	1.31 (0.8 – 2.14)	740 (712 – 769)	<0.001 (40.83)
>20 mg/dL	13.4 (7.8-23.06)	436 (315 – 558)	
Serum Creatinine			
Normal	1.65 (1.1 – 2.66)	729 (699-758)	<0.001 (28.37)
High	13.8 (6.14 – 31.75)	432 (261 – 603)	

ALT, alanine aminotransferase; AST, aspartate aminotransferase; HIV, human immunodeficiency virus; RBC, Red Blood Cells; BUN, Blood Urea Nitrogen; APRI, TSH, Thyroid stimulating hormone; T3, triiodothyronine; T4, Thyroxine; eGFR, estimated glomerular filtration rate.

### Mean (95% CI) for survival time in days and Kaplan-Meier failure function curves

**Figure 4:**
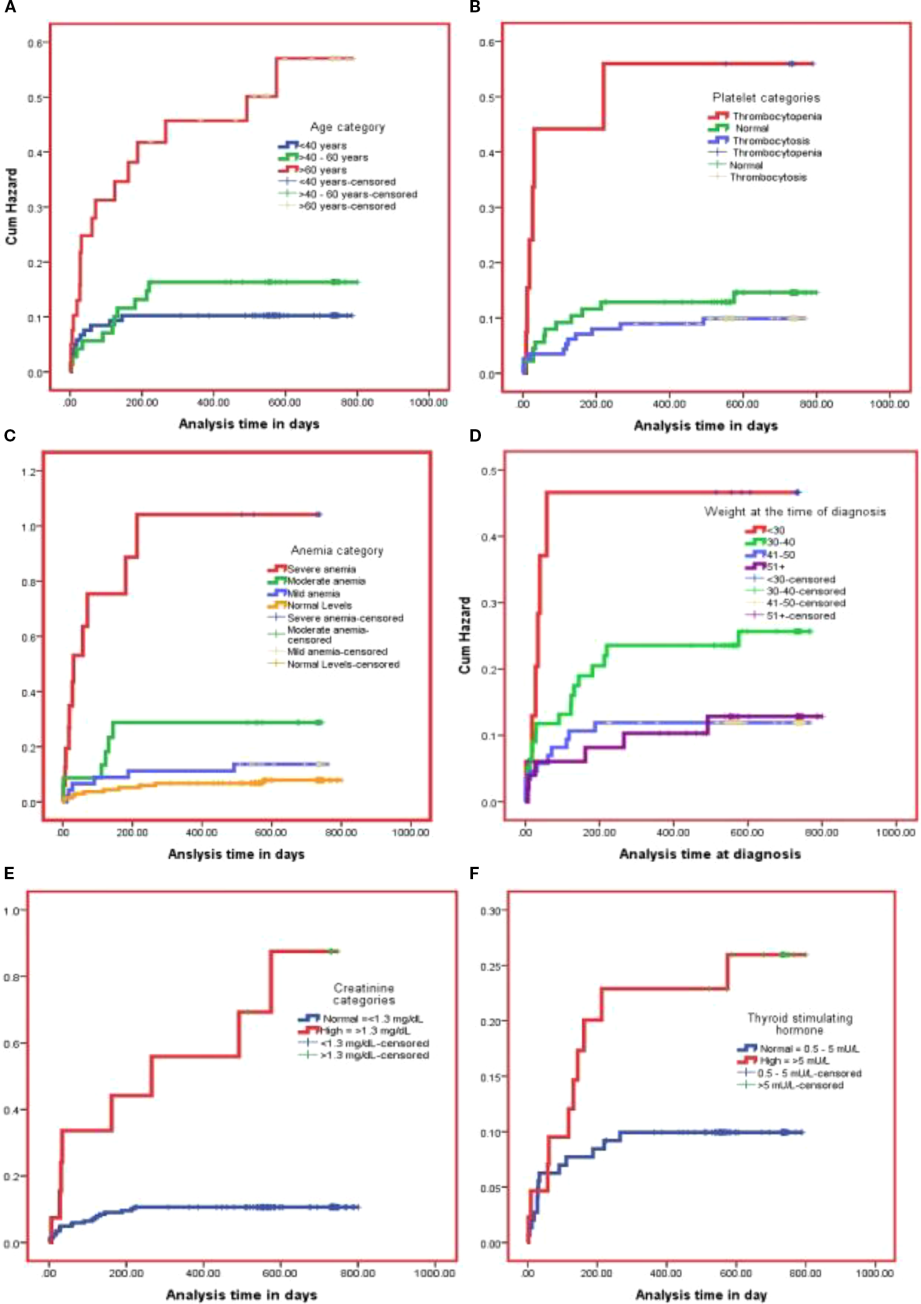
Kaplan-Meier curves for mortality stratified by baseline age **(A)**: Age categories (<40 years, >40–60 years, and >60 years) **(B)** Platelet categories (Low, Normal, High) **(C)** Anemia categories (severe anemia, moderate anemia, mild anemia, normal Hb levels) **(D)** Weight in Kg (<30 kg, 30–40 kg, 41–50 kg > 50 kg), **(E)** Creatinine categories (Normal, high) **(F)** TSH (High TSH, Normal TSH). P < 0.05 for all.

**Figure 5:**
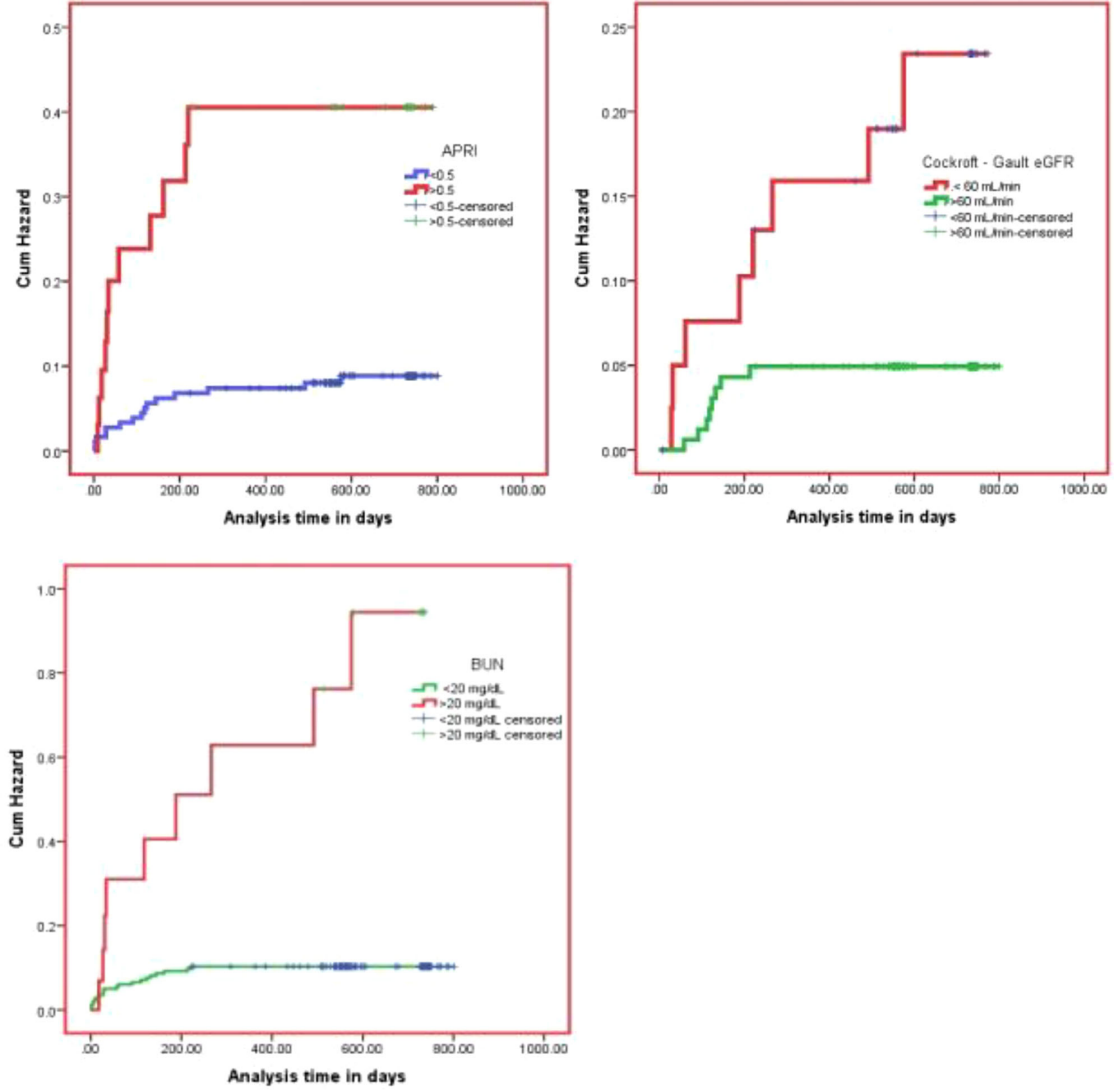
Kaplan-Meier curves for mortality stratified by baseline APRI score categories, Cockcroft-Gault eGFR _Creat_ and Blood Urea Nitrogen (BUN), P < 0.05 for all.

**Table 3** shows mean (95% CI) for MDR-TB patients. According to estimates shown, overall program follow-up duration in the period spanning 2013–2023 was 557.4 days (525 – 589.1 days). The median (IQR) time to death, being cured or completing treatment was 33(IQR: 10-130) days, 731(556-735) days; and 734 (732-740) days, respectively, P<0.001. Estimates of mean (95% CI) follow duration in days differed with respect to the following: age, PLTs, anemia, weight, SCr, TSH, BUN, APRI scores and eGFR _Creat_. In increasing magnitude, variable with the shortest time-to-mortality included severe anemia: 314.1 days (154 – 475.4) days; high SCr: 432 days (261–603 days); elevated BUN: 436 days (315–558 days); weight < 30 kg: 472 days (304–640 days); low PLT: 474(275 – 674.2), among others. Kaplan-Meier (K-M) failure estimates by age, PLTs, anemia, weight, SCr, TSH, BUN, APRI score and eGFR _Creat_ categories are illustrated in **Table 3** (**Figures 4** and **5**).

### Independent predictors of mortality in patients with MDR-TB in Eritrea

**Figure 6:**
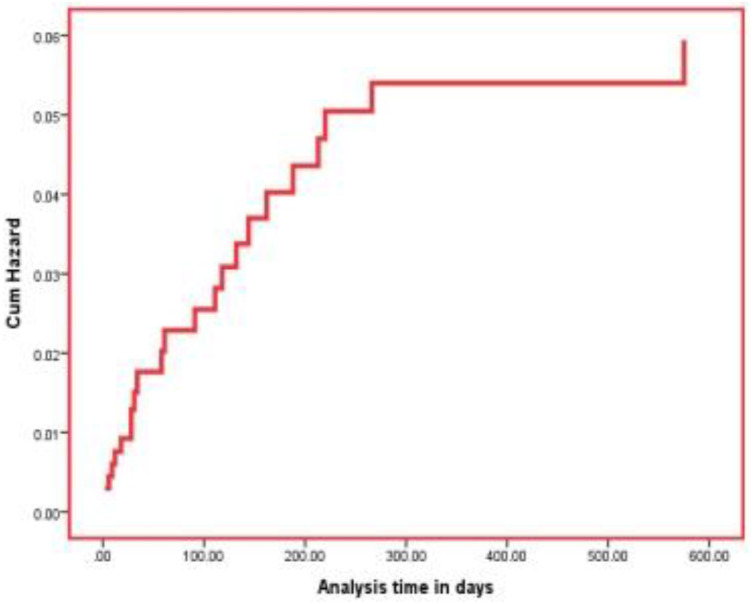
The cumulative incidence probability of mortality for MDR/RR-TB patients in Eritrea, 2013-2023.

Multivariate Cox proportional hazards models evaluating the predictors of 10-year mortality at the National Referral MDR-TB hospital Merhano identified several baseline factors that can affect outcomes (**Table 4**; **Figure 6**). In the adjusted model, we noted that a unit increase in hemoglobin concentration and weight reduced the risk of death by 0.79(95% CI: 0.67 – 0.93), P = 0.005 and 0.96(95% CI: 0.91-1.01), respectively. Further, higher likelihood of death was associated with elevated SCr (>12 mg/dL) levels (aHR = 6.33 (95% CI: 2.33 – 17.19), P<0.001, and APRI Score >0.5 (2.78(95% CI: 1.65 – 4.70), P<0.001. In contrast, a unit increase in age increased the risk of death by 1.045(95 CI%: 1.02 – 1.07), P<0.001 as it can be shown in **Table 4**.

**Table 4:** Cox proportional hazard of mortality among MDR TB patients in Eritrea (2013 – 2023).

Cohort characteristics	Unadjusted HR (95% CI)	P value	Adjusted HR (95% CI)	P-value
Weight at time of diagnosis (Kg)	0.955 (0.91 – 1.01)	0.094	0.96 (0.91-1.01)	0.091
ALT (U/L)				
<40 U/L	1 (Reference)	0.230		
>40 U/L	0.28 (0.3-2.26)			
Thyroid stimulating hormone				
Normal	1 (Reference)	0.395		
Hypothyroidism	1.51 (0.58 – 3.90)			
Serum Creatinine (mg/dL)				
< 12 mg/dL	1 (Reference)	0.004	1 (Reference)	<0.001
>12 mg/dL	5.49 (1.75 – 17.2		6.33 (2.33 –17.19)	
Age at time of diagnosis (years)	1.04 (1.02 – 1.07)	0.001	1.045 (1.02 – 1.07)	<0.001
Platelets count (x10^9^/L)	1.00 (0.99 – 1.01)	0.899		
APRI Score				
<0.5	1 (Reference)		1 (Reference)	
>0.5	3.35 (1.40 – 8.04)	0.107	2.78 (1.65 – 4.70)	<0.001
Hemoglobin	0.78 (0.66-0.926)	0.004	0.79 (0.67- 0.93)	0.005

ALT, Alanine aminotransferase; HR, Hazard Ratio; APRI, Aspartate Amino Transferase platelet ratio index. Hypothyroidism: TSH > 5.

### Impact of treatment on selected clinical chemistry parameters among patients with positive outcomes

In this analysis, significant reductions in specific hemato-biochemical parameters were observed. These included reductions in PLT from 356 x10^9^/L (IQR: 267-461.2 x10^9^/L) to 296 x10^9^/L (IQR: 224.7–374 x10^9^/L) at EOT; WBC from 7.0 x10^9^/L (IQR: 5.0-9.0 x10^9^/L) to 6.0 x10^9^/L (IQR: 4.13-8.0 x10^9^/L) at EOT. Increases in the last measurements at EOT were also observed for T_3_ and T_4_. **Table 5**. In contrast, significant increases were observed in several parameters at EOT. These included increases in BUN from 11.5 mg/dL (IQR: 9–15 mg/dL) to 13 mg/dL (IQR: 10–15 mg/dL); TSH from 2.8 μU/L (IQR: 1.58-5.0 μU/L) to 4.5 μU/L (IQR: 3.0-9.0 μU/L); Hemoglobin from 13.0 g/dL (IQR: 12.0-15.0 g/dL) to 14.26 g/dL (IQR: 13.0-16.0 g/dL); hematocrit from 40.0% (36.0-45.0%) to 43%(39.0-47.0%); and RBC from 5.0x10^6^/µL (4.0-5.0 x10^6^/µL) to 5.10x10^6^/µL (4.0-6.0x10^6^/µL). Finally, the proportion of patients with anemia, hypothyroidism, elevated BUN, elevated SCre, thrombocytosis and APRI > 0.5 at EOT were 4(<1%), 89(43.5%), 24(10.8%); 13(5.9%), 26(117%) and 35(15.3%), respectively. Among patients with hypothyroidism, 44(21.5%) had subclinical hypothyroidism while 45(22%) had Overt hypothyroidism.

**Table 5:** Baseline – vs last measurements before end-of-treatment in specific hemato-biochemical parameters.

Variable	Baseline measurement (Median IQR)	Last measurement (Median IQR)	P-value
ALT (U/L)	14 (8 – 21)	11 (7-18)	0.403
AST (U/L)	29 (23-37)	31 (26-42)	0.189
BUN (mg/dL)	11.5 (9-15)	13 (10-15)	0.028
SCr (mg/dL)	0.7 (0.5 – 0.83)	0.7 (0.6-0.9)	0.012
TSH (μU/L)	2.8 (1.58-5.0)	4.5 (3.0-9.0)	<0.001
Total thyroxine (T_4_) µg/dL	7.95 (6.0-9.1)	6.14 (5.0-8.0)	<0.001
T3 re-uptake	1.0 (0.9-1.1)	1.0 (1.0-1.1)	0.016
Platelets (x10^9^/L)	356 (267-461.2)	296 (224.7-374)	<0.001
Hemoglobin (g/dL)	13.0 (12.0-15.0)	14.26 (13.0-16.0)	<0.001
Hematocrit (%)	40.0 (36.0-45.0)	43 (39.0-47.0)	<0.001
RBC (10^6^/µL)	5.0 (4.0-5.0)	5.1 (4.0-6.0)	<0.001
WBC (x10^9^/L)	7.0 (5.0-9.0)	6.0 (4.13-8.0)	<0.001
APRI	0.23 (0.16 – 0.34)	0.25 (1.64-0.38)	0.706

ALT, alanine aminotransferase; AST, aspartate aminotransferase; HIV, human immunodeficiency virus; RBC, Red Blood Cells; BUN, Blood Urea Nitrogen; APRI, AST/PLT ratio; TSH, Thyroid stimulating hormone; T3, triiodothyronine; WBC, White blood cells; Scr, Serum Creatinine.

## Discussion

Overall, some notable findings associated with this cohort included the absence of XDR-TB in Eritrea; the large number of newly diagnosed MDR/RR-TB cases, 82 (32.4%); the higher proportion of men, 157 (61.1%); the low mean weight at diagnosis, 43.81 (± 10.22) kg; the high mean age (± SD), 41.7 (± 16.5) years; and the large number of patients experiencing ADRs. In many ways, these characteristics are consistent with data from SSA, where men are disproportionately affected. The large number of patients presenting with advanced disease characterized by BMI < 18.5 kg/m² is also a common theme. In Ethiopia, for example, Shimbre et al. reported a male-to-female ratio of 0.75, a median BMI of 15.7 kg/m² (IQR = 12–18.8 kg/m²), and a median age of 27.5 years (IQR = 23–35.5) (Shimre et al., 2020). Comparable findings have been reported in studies from Ghana and Egypt (Sylverken et al., 2021). The predominance of males in MDR/RR-TB clinics in SSA has been attributed to multiple factors, including gender norms which discourage men from admitting ill-health and behavioral risks including alcohol usage, smoking, or drug use in men, among others (Soeroto et al., 2022).

Beyond patient profiles, the main findings of this study were that the proportions of positive treatment outcomes (cured or treatment completed) among MDR/RR-TB patients in Eritrea were relatively high, 207 (80.5%). This result is within the targets recommended by the WHO (75%–90%) and is higher than those reported in Ethiopia and other countries in SSA: Ethiopia was 63.82%–77.12% (95% CI = 73.0–81.5) (Gondar, Ethiopia) (Belachew et al., 2022); 76.32% in Southern Ethiopia (2014–2019) (Kebede et al., 2024); and 62.8% in Central Province, Zambia (Chanda, 2024). In contrast, the treatment success rates are lower than what has been reported in countries with a high HDI (Zhang et al., 2024). In the past, differences in the treatment success rates among MDR/RR-TB patients between low- and high-SDI countries have been linked to multiple factors. These include treatment of patients with a limited formulary, which exposes patients to a greater risk of developing life-threatening ADRs (Perumal et al., 2018); the proportion of patients with XDR; health infrastructure and access; and the availability of quality treatments (novel treatment regimens such as BPaLM and BPaL; adjunctive therapies like immune modulators, therapeutic drug monitoring (TDM), and surgical interventions—e.g., video-assisted thoracoscopic surgery (VATS) for drainage, or extracorporeal membrane oxygenation (ECMO)). Others include social determinants and comorbidities.

On the other hand, intra- and inter-country variation in treatment success rates in SSA countries are mostly due to study designs (closed or open cohorts or reliance on non-standardized questionnaires) (Girum et al., 2018), programmatic factors, and patient-related factors (Umanah et al., 2015). For example, prominent features in Eritrea’s MDR-TB treatment program, such as mandatory institutionalization of MDR/RR-TB patients and the availability of DOTS at the community level, may optimize early diagnosis, limit LTFUs, and improve ATT adherence and overall outcomes. The absence of XDR and the low proportion of patients with HIV/AIDS may also augment successful treatment outcomes.

Further, the magnitude of all-cause mortality was 15.6% (95% CI: 10.5%–19.4%) and the incidence was 2.91 (95% CI: 2.11%–4.11%) per 10, 000 PD. More importantly, a large fraction of deaths occurred within 33 days (IQR: 10–130) after enrollment into care. These results are concordant with previous studies from the region. In their review, Girum et al. reported a prevalence of 12.25% (95% CI: 9.39%–15.11%) (Girum et al., 2018). Relatable estimates have been reported by other workers (Kebede et al., 2024). However, higher death rates have been reported in other cohorts in the region, such as 21.3% in Zambia (Chanda, 2024).

The risk factors most consistently implicated in MDR/RR-TB mortality in SSA include HIV positivity, male sex, presence of comorbidities, and presence of ADRs (Woldeyohannes et al., 2019; Chanda, 2024). Most studies, for instance, report poor outcomes among MDR/RR-TB patients co-infected with HIV, especially those not on ARV or those with suboptimal adherence (Chanda, 2024). Neither of these risk factors was found to be associated with MDR/RR-TB mortality in this cohort. A number of explanations can be invoked to explain these findings. First, MDR/RR-TB settings in SSA are highly heterogeneous, with stark between-study heterogeneity in baseline hazards: program quality, ARV uptake, HIV disease stage, ATT regimen options, access to treatment, and the prevalence of specific comorbidities (Umanah et al., 2015; Soeroto et al., 2022). Other factors which may account for the observed differences include study population bias or confounding by other covariates.

In a separate analysis, we demonstrated that mortality was significantly associated with age > 60 years and weight < 30 kg at baseline. In the Cox regression analysis, a unit increase in age was associated with a 1.045 (1.02–1.07) increase in the likelihood of mortality. Interestingly, the impact of low weight on mortality was attenuated in the multivariate analysis. These associations are well-documented in the literature (Alemu et al., 2020; Oyewusi et al., 2024). A recent review article argued, for instance, that the risk of developing MDR-TB increases with advancing age, particularly among middle-aged and older males in LMICs (Song et al., 2024). Equally well-supported is the nexus between low weight/BMI (a proxy for malnutrition) and adverse outcomes such as delayed culture conversion and mortality (Soeroto et al., 2022). According to most experts, the connection between aging and mortality is largely due to age-related immune senescence, comorbidities, increased susceptibility to ADRs, and differential access to healthcare or differences in health-seeking behavior (Sun et al., 2015). In Uganda, Kizito et al. averred that the inability to afford transport and health facility-based DOT was responsible for suboptimal adherence to treatment in their cohort (Kizito et al., 2021). Although the effect of aging on MDR/RR-TB mortality risk has broad support in the literature, null or contrary findings have been documented (Sun et al., 2015).

In agreement with other studies, we reported a high prevalence of anemia (91; 37.8%) and low median values of hemoglobin, hematocrit, and RBC count. Furthermore, baseline anemia was associated with mortality both in the bivariate and multivariate analysis. The observed association correlated with the magnitude of severity, with severe, mild, and moderate anemia exhibiting the strongest associations. These observations are generally consistent with existing literature. In their paper, Adewole et al. reported a lower mean value for hemoglobin (11.70 ± 2.73 g/dL) in MDR-TB patients (Adewole et al., 2024). In a Brazilian cohort, authors estimated that the prevalence of anemia was 61.2% (27.5% mild, 27.5% moderate, and 6.2% severe) (de Mendonc¸a et al., 2021). In line with previous reports, we found that severe anemia was one of the strongest predictors of mortality in this cohort. In the past, some authors have argued that anemia is a marker of TB or MDR/RR-TB severity and wasting, and is ubiquitous in patients with disseminated and meningeal TB (de Mendonc¸a et al., 2021). Others have further suggested that it is a marker for slower clinical (as well as bacteriological) recovery in response to ATT (Ashenafi et al., 2022).

In general, the etiology of anemia in TB or MDR/RR-TB patients is highly complex. This complexity is readily discernible from the bidirectional nature of causality where anemia can be a risk factor for TB and TB can be a risk factor for anemia (Balepur and Schlossberg, 2016; Cobelens et al., 2021; Gelaw et al., 2021). Furthermore, anemia can be a side-effect of anti-tubercular (ATT) agents such as Lzd, INH, and PAS (Song et al., 2024; Gil-Santana et al., 2019). This causal bi-directionality imposes many difficulties for those trying to untangle the cause of baseline anemia in MDR/RR-TB patients. Although the exact cause of anemia is rarely reported in MDR-TB literature, there is a broad consensus that the most common form is normochromic normocytic anemia associated with chronic disease (Singh et al., 2001). Additional, albeit marginal, contributors include anemia due to metabolic deficiencies (folic acid and B12 deficiency), autoimmune hemolytic anemia (Coombs test positive), and bone marrow complications—such as anemia due to fibrosis, infiltration with granulomas, or miliary/disseminated TB (Balepur and Schlossberg, 2016). Fundamental to these etiologies is a distinct cytokine biosignature characterized by higher levels of pro-inflammatory cytokines (Interleukin-6 (IL-6), Interferon alpha (IFNα), and IFNβ) and profibrotic factors (VEGF, EGF, FGF-2, and PDGF–AB/BB), and lower levels of type-1 cytokines (IFNγ and IL-2) (Dasan et al., 2025).

In addition to anemia, other hematological abnormalities reported in MDR/RR-TB patients include thrombocytosis, leukocytosis, and thrombocytopenia (isolated or as part of pancytopenia), among others (Kirwan et al., 2021). In this cohort, thrombocytosis was present in a large number of patients (53.4%), making it one of the most common abnormalities in this setting. The high prevalence of thrombocytosis is in concordance with estimates from a review by Farhadian and colleagues, who reported a pooled prevalence of 31.9% (95% CI: 15%–55.3%) (Farhadian et al., 2024). The reported upregulation of the PLT transcriptome or elevation of PLT-specific parameters (mean PLT volume (MPV), PLT distribution width (PDW), and plateletcrit) provides additional support for the prominence of thrombocytosis in TB patients (Balepur and Schlossberg, 2016). Regarding causality, the consensus is that the thrombocytosis reported in TB patients is primarily reactive and is driven by inflammatory and infectious processes rather than myeloproliferative disorders (Balepur and Schlossberg, 2016). For this reason, it can resolve with effective ATT.

Although thrombocytosis was a prominent feature in MDR/RR-TB patients at baseline, a significant decline in its prevalence (11.7% or significantly low median values) was reported at EOT. Despite the drastic decline, the condition persisted in a small fraction of patients. The presence of thrombocytosis at EOT should not be dismissed lightly, since it can have significant clinical consequences for patients—including increased risk of venous thromboembolism (VTE), deep vein thrombosis (DVT), and pulmonary embolism (PE). The proportion of deaths attributable to these complications in MDR/RR-TB patients in Eritrea (or SSA) is poorly understood and should be the focus of future studies. Indeed, even the fate of patients with derangements in hematological parameters (e.g., thrombocytosis) who are designated as cured is unknown.

In a related analysis, we demonstrated that a small proportion of patients presented with thrombocytopenia: 14 (5.9%). Interestingly, the incidence of mortality in these patients was relatively high—42.9% of the patients presenting with thrombocytopenia died. A low frequency of thrombocytopenia has been reported by other workers in the region: 8% in Ethiopia (Abay et al., 2018). Although thrombocytopenia is regarded as a rare, therapy-associated hematological disorder in MDR/RR-TB patients, it can have significant clinical consequences (Balepur and Schlossberg, 2016). Literature, for example, connects it to bone marrow infiltration by TB, disseminated intravascular coagulation (DIC), immune thrombocytopenic purpura (ITP), drugs, and thrombotic thrombocytopenic purpura (TTP) (Ozkalemkas et al., 2004; Balepur and Schlossberg, 2016). Another key finding from our study was that thrombocytopenia was a risk factor for mortality in the bivariate analysis but did not remain significant in the multivariate analysis. This may be due to the confounding of thrombocytopenia with other covariates such as anemia—the co-occurrence of the two disorders in pancytopenia is well documented (Balepur and Schlossberg, 2016).

Further, we assessed the relationship between APRI score and mortality in MDR/RR-TB patients. The APRI score, an easy-to-use non-invasive marker for liver fibrosis, has been associated with mortality in liver diseases such as hepatitis B virus or C virus (HCV)-related decompensated cirrhosis (Sarin et al., 2016). However, its role as a predictor of mortality in MDR/RR-TB patients has not been established. This data gap is rather strange considering the fact that high TB burden often co-exists with a high burden of fibrosis-inducing diseases (e.g., chronic HBV, Type 2 diabetes mellitus (T2DM), and malnutrition). More importantly, first-line anti-TB drugs such as INH, RIF, and pyrazinamide (PZA) are known to be hepatotoxic, and their prolonged use can lead to fibrotic changes (Vinikoor et al., 2015).

All told, fibrosis status is an important clinical feature in TB patients but is rarely evaluated at baseline in most SSA clinics. After an extensive search of the literature, we were able to locate two studies where fibrosis status was assessed using the APRI score. In one study, the authors noted that participants with concomitant HIV/HBV/TB had higher APRI scores (Phinius et al., 2020). In the second study, HIV/TB co-infection was associated with an increased likelihood (AOR: 1.18; 95% CI: 1.00–1.39) of fibrosis (APRI score ≥ 1.5) (Vinikoor et al., 2015). In our cohort, an APRI score > 0.5 predicted mortality in 42.3% of cases. More importantly, APRI > 2 (or F4 on METAVIR) predicted death in 100% of the patients. In comparison to other parameters, the APRI score was one of the most important prognosticators of death—more important than PLT count or AST concentrations when considered in isolation. Due to the bidirectional nature of causality between TB and fibrosis/cirrhosis, it was impossible to discern whether fibrosis was present before infection with MDR/RR-TB or the converse. Another important finding was that the median value of the APRI score increased at EOT, with 35 (15.3%) of the patients presenting with values > 0.5. These results suggest a worsening liver fibrosis status and implicate significant drug-induced liver injury (DILI). Considering the limitations of our study (sample size and single measurement), more clinical data—including information on whether individuals had ADRs or acute vs. chronic infections—are needed to understand this finding. Regardless, there is an obvious need to validate non-invasive markers of fibrosis (e.g., APRI score, Fibrosis-4 (FIB-4) index) for use in the country.

Further, we evaluated markers of kidney function including BUN, SCr, and eGFR Creat. Based on these markers, we determined that a significant number of patients had clear evidence of kidney malfunction at baseline: 45 (20.4%) had eGFR Creat < 60 mL/min; 27 (10.5%) had elevated BUN; and 15 (6.4%) had elevated SCr. In general, the prevalence of chronic kidney disease (CKD) in the general population in Eritrea is unknown. Therefore, determining whether these abnormal parameters preceded MDR/RR-TB or were a consequence of TB or ATT (e.g., some drugs used in clinics such as amikacin are nephrotoxic) is difficult. Regardless, previous reports have indicated that reduced eGFR (< 60 mL/min/1.73 m²) can increase the risk of TB, with one study reporting that the aHR of pulmonary TB was 1.45-fold higher in the CKD group than the non-CKD group (Cheng et al., 2018). This risk, Shu et al. reported, may increase with CKD stage, determining for instance that TB incidence increased from stage 3a (HR: 2.40 (1.96–2.96)) to stage 5 (4.85 (3.15–7.65)) and was even higher for patients receiving long-term dialysis (Shu et al., 2020). Further, we demonstrated that elevation in SCr, BUN, and reduced eGFR Creat were significantly associated with mortality. These associations are well established in the literature (Canney, 2023).

Although kidney impairment markers are some of the most important predictors of mortality, the relationship between TB/MDR/RR-TB and CKD in SSA is poorly understood. This is largely due to the limited research on the topic. In countries like Eritrea, where TB notification rates are < 100 per 100, 000 persons per year, latent TB infection (LTBI) screening in high-risk groups—such as anemic or underweight adults, patients with CKD, or those with CKD-predisposing conditions like T2DM—should be implemented. For reference, note that the WHO suggests a focus on high-risk groups in countries where TB incidence is less than 100 per 100, 000 person-years (Getahun et al., 2015).

In patients with MDR/RR-TB, BUN elevation can be linked to factors other than renal impairment. These include cardio-renal or neurohormonal impairments resulting in a hyper-catabolic state and malnutrition; dehydration and volume depletion; and systemic inflammation which can lead to hemodynamic changes and decreased renal perfusion, contributing to pre-renal azotemia (Duan et al., 2023). For these reasons, we found it difficult to assess whether the BUN elevation was due specifically to renal impairments. Regardless, interest in the utility of BUN as a robust marker of mortality in critically ill patients has grown, with some researchers suggesting that BUN levels correlate positively with popular illness severity scores such as APACHE II and SOFA (Arihan et al., 2018). The same authors noted that the association between BUN and mortality in critically ill patients persisted regardless of admission diagnosis and remained significant even after adjustments for confounders in several multivariate analyses. Unfortunately, very few studies have investigated the relationship between BUN elevation and mortality in TB or MDR/RR-TB patients in SSA. In one such study, Yaghi et al. reported that BUN was significantly associated with mortality in TB patients (HR = 1.029 (1.011–1.047)) (Yaghi et al., 2022). In our study, we demonstrated that elevated BUN levels were strongly associated with mortality (e.g., 48% and 62.5% of patients with BUN > 20 mg/dL and 30 mg/dL died). Unfortunately, BUN was not incorporated into the multivariate Cox regression model due to multicollinearity (overlap in explanatory power) concerns. Although BUN was one of the most important predictors of mortality in this cohort, measurements were missing in 25% of patients who died. This should raise concern; in fact, it is our submission that the integration of BUN into standard assessment protocols for MDR/RR-TB management in Eritrea can enhance clinical decision-making.

Last but not least, literature indicates that hypothyroidism is relatively common in MDR/RR-TB. Based on our data, hypothyroidism was present in 45 (21.6%) patients at baseline—15 (7.2%) of whom had overt hypothyroidism—with the number doubling to 89 (43.4%) in the last measurement before EOT, where 45 (22%) had overt hypothyroidism. Various studies have reported hypothyroidism rates ranging from 3.5% to 69%: India (3.9%) (Akshata et al., 2015); Ethiopia (19.8%) (Biranu et al., 2021); Egypt (39.5%) (Ibrahim et al., 2012); and Lesotho (69%) (Satti et al., 2012). These wide-ranging numbers attest to the fact that the exact incidence of hypothyroidism during MDR/RR-TB treatment is unknown and should be the subject of future studies. Literature, at present, implicates a number of ATT agents in the etiology of hypothyroidism in MDR-TB patients. Prominently mentioned in this regard is exposure to second-line ATT agents, including goitrogens such as thiamides (Ethionamide/Prothionamide) and PAS. First-line agents include high-dose INH and RIF (Seung et al., 2015). In our cohort, the doubling of patients with hypothyroidism or median values of TSH at EOT implicates drug-induced hypothyroidism. Of note, PAS and Eto are part of the formulary in this setting. Equally worrisome is the fact that hypothyroidism has vague and non-specific symptoms which can easily be missed by clinicians. Unrecognized, hypothyroidism may lead to adverse outcomes such as increased risk of treatment failure, prolonged recovery, and death. In this cohort, hypothyroidism was associated with mortality in the bivariate analysis; however, the relationship was attenuated in the multivariate analysis. In all, testing for hypothyroidism per guidelines should be emphasized.

### Strengths and limitations of the study

MDR-TB patients from LMICs are often subject to less stringent clinical and laboratory monitoring during treatment. This restricts the information that can be provided in the numerous retrospective studies from the region. In Eritrea, data on a large number of hemato-biochemical parameters is generally available. Leveraging this data, we were able to explore the relationship between these parameters and mortality among MDR/RR-TB patients. Further, novel composite measures such as APRI score and eGFR Creat were evaluated. Although this study has several strengths, a number of limitations must be acknowledged. First, data collection relied solely on patient records/registers, constrained by the information documented by data clerks or clinicians. This prevented us from evaluating other TB transmission factors like level of education, income, family history of TB, serum albumin, height, lifestyle (smoking, alcohol use), time-to-culture conversion, pulmonary consolidation, or the presence of cavitary lesions on chest X-rays. Further, long-term mortality following discharge from the clinic was not evaluated. Several strategies can be implemented to address these limitations in future studies. Expanding data collection sources beyond patient records—such as including data collected by DOTS providers or conducting interviews with patients—can capture critical information which may not be routinely documented in clinical records.

## Conclusion and recommendations

Accurate determination of factors associated with mortality in MDR/RR-TB patients is critical for patient management and population-level interventions. Using information generated from this study, clinicians from the region can better stratify the mortality risk of patients admitted to the MDR-TB hospital by recognizing key mortality risk factors. Overall, we uncovered a steady decline in the incidence of MDR/RR-TB in Eritrea. However, mortality rates have declined at a slower rate than the observed decline in incident TB or MDR/RR-TB. Further, mortality rates were relatively low (15.6%, 95% CI: 10.5%–19.4%), with a significant fraction of deaths occurring within 33 days. The large number of deaths occurring within days or weeks and the significant number of patients presenting with markers of disease severity (low weight, severe anemia) point to the possibility that late diagnosis of MDR/RR-TB is a problem. To ameliorate this, proactive strategies are needed to improve early detection of MDR/RR-TB. Improvements in supportive care for patients with severe complications should also be prioritized. In increasing magnitude, variables with the shortest time-to-mortality included severe anemia, high SCr, elevated BUN, weight < 30 kg, low PLT, high APRI score, and hypothyroidism. In the multivariate Cox regression analysis, independent predictors of mortality included weight, SCr, age, hemoglobin levels, and APRI score. Going forward, additional research will be needed to clarify some of the issues raised in this study.

## Data Availability

The original contributions presented in the study are included in the article/supplementary material. Further inquiries can be directed to the corresponding author.
